# Effects of Sirolimus on Lung function in patients with Lymphangioleiomyomatosis

**DOI:** 10.22088/cjim.10.1.7

**Published:** 2019

**Authors:** Fatemeh Aghaeimeybodi, Katayoun Najafizadeh, Seyd-Kazem Razavi-Ratki, Nasim Namiranian

**Affiliations:** 1 Department of Internal Medicine, Shaheed Sadoughi University of Medical Sciences, Yazd, Iran; 2 National Research Institute of Tuberculosis and Lung Disease, Masih Daneshvari Hospital, Tehran, Iran; 3 Department of Radiology, Shaheed Sadoughi University of Medical Sciences, Yazd, Iran; 4 Yazd Diabetes Research Center, Shaheed Sadoughi University of Medical Sciences, Yazd, Iran

**Keywords:** Sirolimus, Lymphangioleiomyomatosis, Lung function

## Abstract

**Background:**

Lymphangioleiomyomatosis (LAM) is a progressive lungs disease that affects women at reproductive years. Sirolimus inhibits mammalian target of rapamycin (mTOR) and its administration in past studies was hopeful in treatment of patients with LAM. The aim of this study was to evaluate sirolimus therapy on lung function in LAM patients.

**Methods:**

We conducted a trial to evaluate the effect of sirolimus on six patients with LAM who had severe or very severe obstructive lung disease, and one-year follow up. Maintenance level of Sirolimus was 10-15 ng/ml. Serial visits (including physical examination, evaluation of signs and symptoms of disease and adverse events due to treatment), spirometry, 6MWT done at baseline 3, 6, 9 and 12 months after. Spirometric parameters walk distance and O_2_ saturationbefore and after exercise at first and the end of treatment were measured.

**Results:**

Four patients had TSC-LAM while the other 2 patients had S-LAM. The mean level of sirolimus was 13 ng/ml after one-year treatment. Mean FEV1 at enrollment and end of study was 1000cc (33% predict) and 1228cc (42% predict) respectively (P=0.674). The mean FVC at baseline and end of study was 1648cc (49% predict) and 1866cc (55% predict) (P=0.996). The mean FEV1/FVC at enrollment and the end of treatment was 58% and 62% respectively (P=0.753). The mean FEF25-75 at first and at the end of treatment was 16% and 26%, respectively (P=0.028). The mean walk distance in 6MWT at first and at the end of study was 315 meters (P=0.9). The mean percentage of O_2_ saturation at rest was 84% and 92% at first and at the end of study (P=0.104).

**Conclusion:**

In LAM patients, sirolimus has been shown stabilizeto or improve lung function, rest and exertional O_2_ saturation. Sirolimus was effective in LAM patients who had severe or very severe physiological disorders.

Lymphangioleiomyomatosis (LAM) is an uncommon neoplastic and systemic disease. LAM occurs sporadically (S-LAM) or with tuberous sclerosis complex (TSC–LAM) (1). 

It is associated with lung cystic destruction, recurrent pneumothoraces, abdominal tumors and chylous effusions in thorax and abdomen (2, 3). LAM affects young women almost exclusively in reproductive years. LAM involves all races. Prevalence of disease is 2.5 per million. Prevalence of tuberous sclerosis complex is 40 per million. Respiratory manifestations occur in 5 to 20% of patients with TSC. The TSC-LAM is 10 times more prevalent than S-LAM (1, 4). Sirolimus in rodent models of TSC made neoplastic growth regression in the liver and kidney (4-6).

Sirolimus blocks hyperphosphorylation of s6 resulting in regular cell growth. Sirolimus side effects are oral aphthous, hypercholesterolemia, pneumonia, diarrhea, cellulitis, pyelonephritis, headache, palpitation, mucositis and etc (2-11). The aim of this study was to evaluate sirolimus therapy on lung function in LAM patients.

## Methods

We conducted a before-after clinical trial of sirolimus on 8 LAM patients in Masih Daneshvari Hospital, Tehran. The study samples were rapid progressive S-LAM or TSC-LAM. Patient’s diagnosis was on the basis of biopsy, clinical findings, HRCT and TSC presentation. The patients with each of the following criteria were excluded: current or planned pregnancy, breast feeding, plural effusion, cardio-pulmonary transplantation, liver dysfunction (AST or ALT>150u/l, Albumin>150 g/lit), hematologic dysfunction (HCT<30%, plt<10000/mm, neutrophil<1500/cc, WBC<3000/mm), renal hemorrhage in past year, proteinuria> 1gr/24hrs, recurrent infection, current surgery in past 2 months, known as sirolimus allergy, creatinine>2.5mgr/dlit, malignancy or stroke or myocardial infarction history, estrogen medication and a patient who was not able to do pulmonary function test (PFT) or six-minute walk test (6MWT). Although LAM cases are rare, we used convenient non-randomized sampling and to increase the power of the study, before-after method was performed. Informed consent was obtained from each subject and sirolimus adverse effects were explained to each patient. The study was approved by the Medical Ethics of Shahid Beheshti University of Medical Sciences. 

**Sirolimus therapy and follow up: **The target sirolimus blood level of 10-15 ng/ml, and 12 months follow-up was considered. Oral 2mg sirolimus was administered and follow-up visits were conducted every three months. The following assessment was done in every visit: PFT including statistic, dynamic, volume and flows and FEV1/FVC, FEV, FVC, FEF25%-75%. The distance covered in the 6MWT: the distance and O_2_ saturation at the baseline and the end of study was checked. Clinical presentation such as cough, chest pain, dyspnea, pneumothorax, chilothorax, medication side effects, lipid profile and complete blood count (CBC) were checked.

**Statistical analysis:** Statistical analyses were done by SPSS Version.19). The LAM patients’ improvement was defined as the mean differences of FEV1, FVC, FEV1/FVC, FEF25%-75%, distance and O_2_ saturation in 6MWT before and after sirolimus treatment. The non-parametric paired t-test (Wilcoxon rank test) was used. Because of limited sample size (8 patients) and non-symmetric distribution, data were presented as mean ± standard error of mean (SEM) and range (min-max). 

## Results

A total of 8 LAM patients (4 TSC-LAM and 4 S-LAM) were eligible for the study. Five patients were diagnosed based on biopsy (3 open biopsy and 2 TBLB) and 3 patients on CT-scan and TSC presentation. One of the cases died while the other underwent lung transplantation. Finally 6 patients completed the study with 12 months follow up. The mean age of patients was 34.21 years old (range: 26-50). Pneumothorax was the primary event leading to diagnosis in 2(33%) cases, cough and dyspnea in 4(67%) patients. All cases were treated with medroxy-progesterone and bronchodilators. None of them were smokers. The patient's PFT at the enrollment was severe obstructive in 4 cases and very severe obstructive in 2 cases. The blood mean level of sirolimus was 13 ng/dl. The impact of sirolimus on PFT, 6MWT distance and O_2_ saturation was illustrated in tables 1-2. 

**Table 1:** Pulmonary function test and 6 minute walk distance characteristics in 6 LAM patients before and after sirolimus treatment

**PFT/6MWT**	**Baseline**	**After 12 months**	**Mean ± SEM (range)**	**P-value**	**Power**
FEV1/FVC (%)	58 ( 35-83)	62 (42-74)	4±19	0.753	0.19
FEV1 (%)	33 (16-58)	42 (14-75)	9±17	0.674	0.18
Mean FEV1 (cc)	1000	1228	228±471	0.753	0.25
FVC (%)	49	55	6±24	0.916	0.22
Mean FVC (cc)	1648	1866	218±785	0.917	0.23
FEF 25%-75% (%)	16	26	10±24	0.028	0.7
Mean of distance 6MWT	305 (234-398)	315	10	0.9	0.76
O_2_ saturation in start of 6MWT (%)	84	92	8±11	0.104	0.57
O_2_ saturation at the end of 6MWT (%)	74	79	5±10	0.272	0.39

**Table 2:** Pulmonary function test and 6-minute walk distance characteristics in 6 LAM patients during 12 month sirolimus treatment

**PFT/6MWT**	**Baseline**	**After 3 months**	**After 6 months**	**After 9 months**	**After 12 months**	**p-value**
FEV1/FVC	0.33±0.167	0.583±0.131	0.598±0.109	0.615±0.111	0.625±0.110	0.753
FEV1	0.336±0.167	0.365±0.191	0.408±0.217	0.428±0.111	0.625±0.110	0.674
Mean FEV1	1000±516.2	1133.3±637.6	1047±594.7	1236.6±697.8	1228.3±771.3	0.753
FVC	1648.3±547.8	1841.6±696.9	1743.3±709.6	1833.3±785.3	1866.6±919.6	0.916
FEF 25%-75%	0.165±0.102	0.201±0.13	0.196±0.117	0.191±0.076	0.26±0.165	0.028
Mean of distance 6MWT	305±68.84	292.5±135.09	343.33±124.17	369.16±139.67	315.16±130.57	0.9
O2 saturation in start of 6MWT	0.843±0.11	0.863±0.07	0.886±0.04	0.871±0.06	0.928±0.02	0.104
O_2_ saturation at the end of 6MWT	0.745±0.14	0.768±0.13	0.775±0.085	0.783±0.10	0.79±0.116	0.272

**Figure 1:**
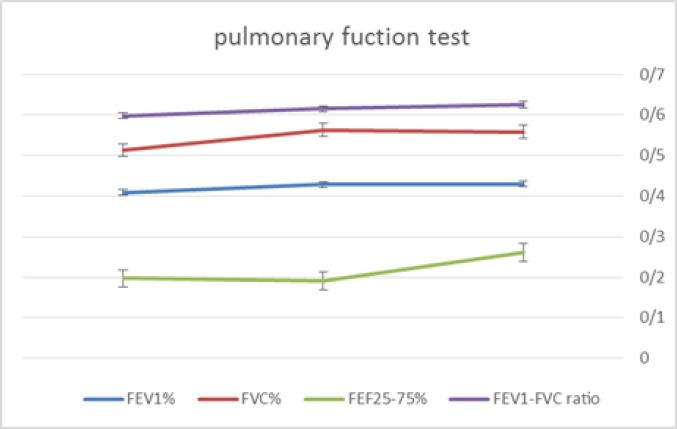
Pulmonary function test improvement during 12 months

Also, the improvement pattern of PFT was shown in figure 1. Cough and dyspnea improved clinically in all patients. Adverse events occurred as follows: Aphthous ulcer in 3 cases, peripheral edema in 1 case, headache in 2 cases and oral abscess in 1 case. Respiratory infections and sirolimus pneumonitis were not reported. One of the six patients stopped sirolimus treatment after 12-month follow-up period because of recurrent headache.

## Discussion

In our study, the mean of FEV1 and FVC increased by 228cc and 218cc after one-year treatment with sirolimus, respectively. The mean FEV1 and FVC at baseline were 1li (33% pred) and 1.648li (49% pred), respectively. Two patients were diagnosed with severe obstructive disease and four patients had severe obstructive disease. In a study by Neurohr et al. in 2011 on 10 female patients with LAM, the mean gain of FEV1 and FVC was 345cc and 448cc after 6 months treatment with sirolimus. The mean FEV1 and FVC at baseline were 36% pred and 69% pred, respectively. The maintenance level of sirolimus was 5-10 ng/ml in their study (9). In a study conducted by McCormack et al. in 2011, the mean gain of FEV1 and FVC was 153cc and 226cc after 12 months of treatment, respectively (8). Their trial involved 89 patients with LAM who had moderate lung impairment and sirolimus levels in the active –treatment group were maintained between 5 and 15ng per milliliter. Maintenance level of sirolimus in our study is in line with two other studies. Our study patients had more severity of pulmonary dysfunction and our finding demonstrated a comparable gain of FEV1 and FVC with other studies. However, because of the small sample size in our work, the sirolimus effect was not statistically significant .Another possible explanation for the lack of a significant effect of sirolimus may be the severity of disease in the studied patients.

In our study, 50% of patients had FEV1 values above baseline and 33% of patients had FVC values above baseline values. Frequency of patients with FEV1 values above baseline in our study is comparable with the study of McCormack et al. (50% vs. 46%). Frequency of patients with FVC values above baseline in McCormack et al. (8) study was 54% that is more than our study that may be explained with severity of disease in our patients. Our findings suggested that sirolimus may be useful in patients with LAM and severe pulmonary dysfunction. Patients of our study revealed a modest increase in 6MWT at 12 months that was not statistically significant.

In the study of Neurohr et al. (9), however, increase in 6MWT at 6 months was 49 meters but was not significant because increasing of distance in 6MWT up to 50 meters increase in 6MWT is considered as normal. So, this value in McCormack et al.’s study was not significant as well. As the strength of our study, we recorded O2_sat_ at baseline and after 6MWT, revealed 8% and 5% gain at the end of treatment. It may be related to the improvement of air flow and V/Q matching during walking that leads to better exertional activity of patients. FEF 25-75% as a measure for evaluating of small airway function was recorded in our study after one year treatment with sirolimus that revealed a significant increase as compared with baseline value. This value is not recorded in other studies. Effects of sirolimus on smooth muscles of small airways lead to significant improvement of air flow.

In conclusions the present study demonstrated that sirolimus administration in Iranian patients with LAM can improve FEV1, FVC and oxygenation. Nevertheless, this improvement is not statistically significant. Future studies with large sample sizes are recommended to better clarify the effect of sirolimus on LAM. Nonetheless, sirolimus improved air flow of Iranian patients with LAM, it is better that these results be confirmed by a more sensitive test e.g. cardiopulmonary exercise test (CPET). Furthermore, sirolimus is relatively safe in Iranian patients with LAM.
